# Selective extraction of scandium from other rare earth elements using a hydrophobic protic ionic liquid composed of decanoic acid and TOA

**DOI:** 10.55730/1300-0527.3745

**Published:** 2025-06-26

**Authors:** Pius Dore OLA, Reo NISHIOKA, Yoshiro TAHARA, Michiaki MATSUMOTO

**Affiliations:** 1 Department of Chemistry, Faculty of Science and Engineering, Nusa Cendana University, Kupang, Indonesia; 2 Department of Chemical Engineering and Material Science, Faculty of Science and Engineering, Doshisha University, Kyoto, Japan

**Keywords:** Selective extraction, scandium, rare earth elements, protic ionic liquid

## Abstract

Rare earth elements (REEs) are increasingly being used in advanced materials, but their extraction remains a major challenge. This is primarily due to their nearly identical ionic radii and similarity in chemical and physical properties. Therefore, this study aims to explore the use of a hydrophobic protic ionic liquid (PIL) composed of decanoic acid and trioctylamine (TOA) to selectively extract scandium (Sc) from other REEs using acid or salt as the extraction medium. In the acidic medium, the extraction efficiency of all tested REEs was inversely proportional to the acid concentration. Meanwhile, the extraction percentage of Sc was significantly higher than that of other REEs in the HCl medium, showing that the condition could be used to selectively extract Sc. The results showed that HNO_3_ medium led to a higher overall extraction percentage but lower selectivity among REEs. The extraction efficiency of REEs in salt media was directly proportional to salt concentration. At higher salt concentrations, the extraction efficiencies became similar, suggesting that the condition could be used for collective extraction. At an organic-to-aqueous phase volume ratio of 0.7, Sc was fully extracted, and dysprosium extraction remained below 17%, offering a promising approach for the selective extraction of Sc from advanced materials and addressing a key challenge in REE separation.

## 1. Introduction

Over the years, there has been a considerable increase in the use of rare earth elements (REEs) in advanced materials. The applications of REEs can be categorized into three main areas: clean energy (e.g., lighting, batteries, wind power), defense (e.g., optics, lasers, aircraft materials, power and communication tools, solar transducers), and high-tech and everyday devices (e.g., hybrid vehicles, LCD screens, medical use, cell phones, CDs, DVDs) [1]. Although the term “rare earth metals” is often misleading, some REEs are more abundant in the Earth’s crust than other precious metals [2]. REEs can occur together in the same deposits because of similarities in their chemical and physical properties, posing a challenge for their extraction [3–5]. This challenge has intensified studies into their selective extraction.

Solvent or liquid-liquid extraction is an effective method for extracting REEs because it is straightforward, rapid, and versatile across a range of concentrations [5,6]. In this method, some metals in the aqueous phase can coordinate with the oxygen atoms of an extractant in the organic phase to form a complex compound, resulting in the transfer of REEs from the aqueous phase to the organic phase [7]. Therefore, the extractant plays a crucial role in achieving both high efficiency and selectivity in solvent extraction. There are six kinds of extractants, namely carboxylic acids, amines, ionic liquids (ILs), chelating agents, organophosphorus compounds, and synergistic extractants [7,8]. Among all these, organophosphorus extractants are commonly used in industry for extracting REEs due to their effectiveness [7]. Phosphorus-based extractants are also gaining significance in industrial extraction to meet the continuous global production needs. For instance, production increased to 210,000 t in 2019, marking an 11% rise over the level in 2018 [9]. Despite the potential, phosphorus-based extractants pose environmental hazards, have small separation factors, can cause stripping difficulties, require complex synthesis processes, and cannot remove all impurities [10].

In recent years, the use of ionic liquids (ILs), which are liquid chemicals at room temperature and are composed solely of ionic species, as REE extractants has attracted significant attention. ILs can be considered “green” due to their ability to reduce or prevent pollution and waste at the laboratory and industrial scales [11]. Several ILs have been applied in REE extraction, such as tri-n-octylmethylammonium (2-sec-octylphenoxy) acetate ([N1888][SOPAA]) [12], tri-n-octylamine and octanoic acid [13], and trihexyltetradecylphosphonium 3-hydroxy-2-naphthoate [14]. Despite the potential, their synthesis requires several steps, including quaternization, anionic exchange, and purification [15]. Consequently, attention has shifted toward protic ionic liquids (PILs) for use as extractants.

PILs are a subclass of ILs with similar properties but can be prepared through a proton transfer from a Brønsted acid to a Brønsted base [16]. In practice, PILs can be synthesized via dropwise addition of the Brønsted acid to the Brønsted base until stoichiometric neutralization is achieved, using a combination of manual and automated methods [17]. Consequently, the three-step-process of aprotic IL synthesis namely quaternization, anionic exchange, and purification [15] is reduced to a single step for PIL preparation. A previous study [18] used a PIL composed of decanoic acid and TOA to recover neodymium (Nd), dysprosium (Dy), and nickel (Ni) from aqueous acid (HCl and HNO_3_) media and aqueous salt (NaCl and NaNO_3_) media. The results showed that the salt medium resulted in a higher extraction percentage compared to the acid medium due to differences in the extraction mechanism. The previous study also showed that Nd and Dy can be separated from Ni. The challenge of extracting REEs is caused by the coexistence of different REEs in the same deposit. Therefore, this study aims to selectively separate Sc from other REEs using a PIL as an extractant in acidic media (HCl and HNO_3_) and salt solutions (NaCl and NaNO_3_).

Sc, a common REE, is widely distributed throughout the Earth’s crust, in more than 100 Sc-bearing mineral species [19]. The main usage of Sc is in the production of aluminum-scandium alloys, which are composed of 2 wt% [20]. Compared to traditional high-strength alloys, Al-Sc alloys offer superior strength, high resilience, refined grain structure, exceptional corrosion resistance, and reduced risk of hot cracking during welding [21]. In addition, the element is used in contemporary fields, such as analytical standards, electronic parts, high-intensity metal halide lamps, fuel cells, and as oil well tracer [22]. Due to the importance of Sc, every effort to selectively extract it from the other REEs is indispensable. Several studies have been carried out to selectively extract Sc from diverse sources and have shown notable extraction efficiency [22–27]. However, these studies used phosphorus-based extractants. Although PIL has been studied for REEs separation [28], there is no information on the use of a PIL comprising only decanoic acid and TOA for the selective extraction of Sc. Compared to the commonly used phosphorus-based extractants or aprotic ionic liquids, this PIL not only has a simpler preparation process but also exhibits a lower viscosity, eliminating the need for dilution during solvent extraction.

## 2. Materials and methods

### 2.1. Chemicals and instruments

Decanoic acid (≥98%) and dodecanoic acid (≥ 98%) were obtained from (Fujifilm-Wako Pure Chemical Corp., Osaka, Japan). N,N,N-trioctylamine (TOA, ≥ 98%) and trihexylamine (THA, ≥98%) were purchased from (Tokyo Chemical Industry Co. Ltd., Tokyo, Japan). All the chemicals used in this study were of analytical grade and were used without purification. Infrared (IR) spectra were recorded over the range of 400 to 4000 cm^−1^ using a Fourier transform infrared spectrometer (FTIR) (Shimadzu IRAffinity-1, MIRacle 10; Shimadzu Corp., Kyoto, Japan) with the attenuated total reflectance (ATR) method. A rotary E-type viscometer (TV-35; Toki Sangyo Co. Ltd., Tokyo, Japan) was applied to measure the viscosity of the PIL. Inductively coupled plasma atomic emission spectroscopy (ICP-AES) (ICPS-8100; Shimadzu Corp., Kyoto, Japan) was used to determine the concentration of REEs.

### 2.2. Preparation of PIL

Preparation of PILs was conducted according to the procedure reported previously [18]. Dodecanoic and decanoic acids were liquefied at 60 °C, and equimolar amounts of liquid alkyl carboxylic acid and trialkylamine were taken in a 300 mL Erlenmeyer flask, then stirred for 4 h at room temperature until a clear liquid formed. To confirm the formation of PIL, Fourier transform infrared spectroscopy (FTIR) (Shimadzu IRAffinity-1, MIRacle 10; Shimadzu Corp., Kyoto, Japan) was performed using the ATR method. The viscosity of each PIL was measured in triplicate with a rotary E-type viscometer (TV-35; Toki Sangyo Co. Ltd., Tokyo, Japan).

### 2.3. REE extraction procedure

Solvent extraction of REEs started with the preparation of the organic phase and aqueous phase. The synthesized PILs were directly used as the organic phase, without any dilution at any stage, while the aqueous phase was prepared by dissolving a metal solution (10 mmol dm^−3^) in hydrochloric acid, nitric acid, sodium chloride, and sodium nitrate in several molar concentrations, without any pH adjustment or control. Metal extraction was performed by mixing an equal volume (2 mL) of PIL and aqueous solution, and shaking the mixture at room temperature using a vortex mixer (1000 rpm). After shaking, the mixture was allowed to stand for >12 h to allow phase separation. The concentration of metal in the aqueous phase was measured using inductively coupled plasma atomic emission spectroscopy (ICP-AES, ICPS-8100, Shimadzu, Kyoto, Japan). Therefore, the percentage of extraction was calculated using Equation (1).


(1) $$\%Extraction=\frac{{\left[M\right]}_{IL.eq}}{{\left[M\right]}_{aq.int}}\times 100=\frac{{\left[M\right]}_{aq.int}-{\left[M\right]}_{aq.eq}}{{\left[M\right]}_{aq.int}}\times 100$$

where [*M*]*_aq.eq_* and [*M*]*_aq.int_* are the equilibrium and initial concentrations of the metal in the aqueous phase, respectively. The values obtained were averaged over three measurements.

## 3. Results and discussion

### 3.1. Characterization of PIL

**Figure 1:**
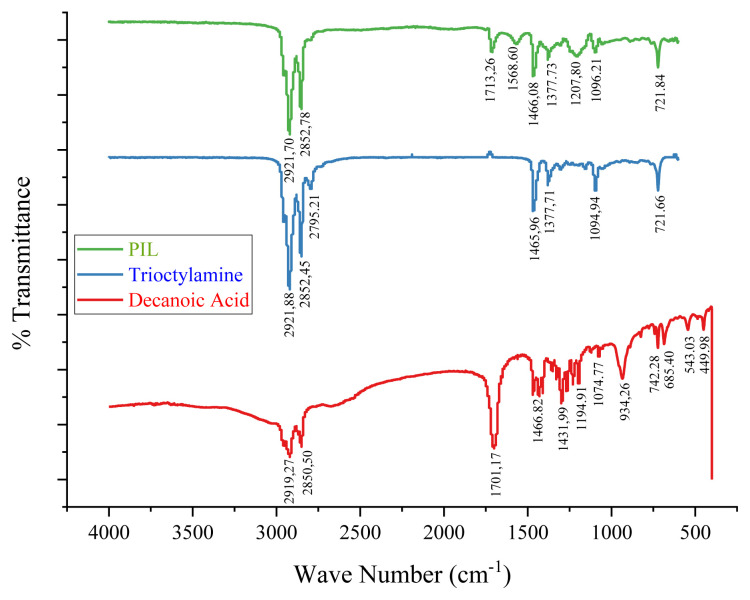
Fourier transform infrared spectra of PIL, decanoic acid, and TOA.

FT-IR spectra of prepared PIL, decanoic acid, and TOA are shown in Figure 1. From the FTIR spectra, a characteristic band in the spectrum of decanoic acid appears at 934.26 cm^−1^, corresponding to the out-of-plane bending of the −OH group, which typically occurs at 910–950 cm^−1^ [29]. In the PIL spectrum, this peak is absent due to deprotonation, forming a carboxylate anion (COO^−^), which is confirmed by the emergence of a new peak at 1568.60 cm^−1^. This peak typically occurs in the range 1550–1610 cm^−1^, attributed to the asymmetric stretching of the COO^−^ [30]. There is a peak at approximately 1200 cm^−1^ in the PIL spectrum, but not in decanoic acid or TOA. This peak is presumably due to the C–N stretching of the protonated amine group (HR_3_N^+^), which is generally observed at 1050–1210 cm^−1^ [31]. A medium peak at approximately 1094 cm^−1^ in the TOA spectrum, due to C–N stretching of the tertiary amine (R_3_N), which commonly occurs in the 1020–1120 cm^−1^ region [29], disappears in the PIL spectrum, indicating that the tertiary amine has been protonated to form HR_3_N^+^. The peaks appeared around 1400 cm^−1^ in all three spectra derived from symmetric and asymmetric bending of −CH_2_ and −CH_3_, which are typically observed in the regions of 1370–1390 cm^−1^ and 1450–1470 cm^−1^, respectively. The stretching of these groups is observed near 3000 cm^−1^ [29]. A strong peak of approximately 1700 cm^−1^ observed in decanoic acid and PIL spectra results from the presence of the carbonyl functional group, which is absent in TOA. Overall, interpretation of these functional group signals confirms deprotonation of the carboxylic acid to yield RCOO^−^ and protonation of TOA to produce the cation HR_3_N^+^. The PIL is formed through the attractive force between the cation and anion, as indicated in the scheme below:

Conventional aprotic ILs were highly viscous, which affected mass transfer in the solvent extraction system. For instance, the viscosities of [emim][BF_4_], [emim][Tf_2_N], and [emim][OAc] were 44.89, 39.19, and 181.06 mPa•s, respectively [18]. Therefore, ILs with lower viscosity need to be developed to enhance extraction efficiency. PILs were synthesized from pairs of alkyl carboxylic acids (dodecanoic, decanoic, octanoic, and heptanoic acids) and trialkylamines (TOA and trihexylamine [THA]) to study the effect of chemical structure on viscosity—a key parameter influencing efficient mass transfer. The acids and amines were selected due to their hydrophobic nature. As presented in Table 1, PILs synthesized possessed viscosities of 5.65 to 20.90 mPa•s (at 30 °C), considerably lower than common aprotic ILs. PILs derived from TOA exhibited higher viscosities than PILs synthesized using THA, and PILs prepared with shorter-chain acids showed lower viscosities than those with longer-chain acid. These results emphasized that the rational design of PILs through acid and amine choice could provide low-viscosity solvents, facilitating mass transfer and improved extraction kinetics, leading to more efficient and convenient solvent extraction operations.

Preliminary copper extraction studies were conducted using these PILs. The extraction efficiency of TOA was higher than that of THA. Meanwhile, carboxylic acids had little effect on copper extraction efficiency. In subsequent experiments, the PIL composed of TOA and decanoic acid, which are commonly used as extractants, was investigated.

### 3.2. REE extraction using PIL

#### 3.2.1. Effect of time

**Figure 2:**
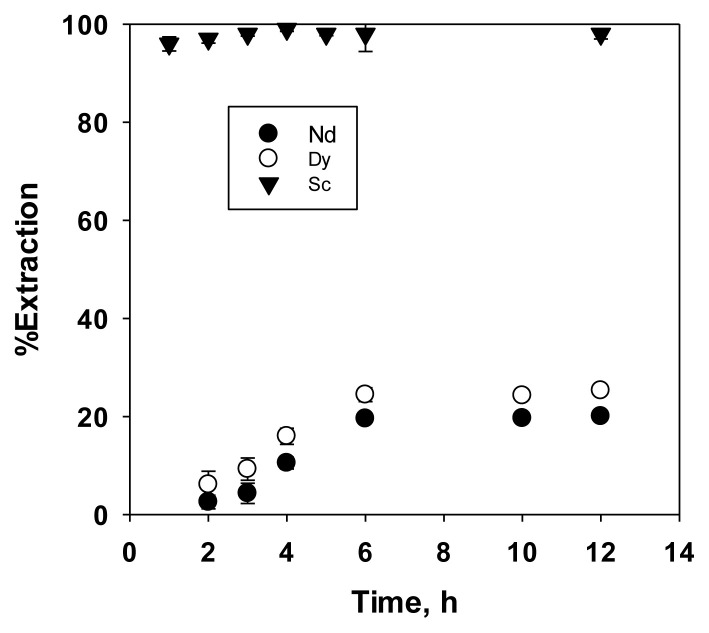
Effect of time on the extraction of Sc, Dy, and Nd using PIL as the extractant.

The first step of this study was to investigate the time needed to reach extraction equilibrium of 10 mmol dm^−3^ metals in 0.1 mol dm^−3^ hydrochloric acid, as shown in Figure 2. In this study, the time to reach extraction equilibrium for Sc was shorter (3 h) than that for Nd and Dy (6 h), with a significantly higher extraction efficiency (100%) than that for Nd and Dy (approximately 20%). The faster and more efficient extraction of Sc compared to Nd and Dy showed a stronger affinity of Sc for the PIL, attributed to its higher charge density resulting from its lower ionic radius relative to Nd and Dy [27]. A similar experiment was conducted to assess the extraction selectivity of Sc compared with other REEs after 6 h of shaking.

#### 3.2.2. Effect of acid concentration on REE extraction

**Figure 3:**
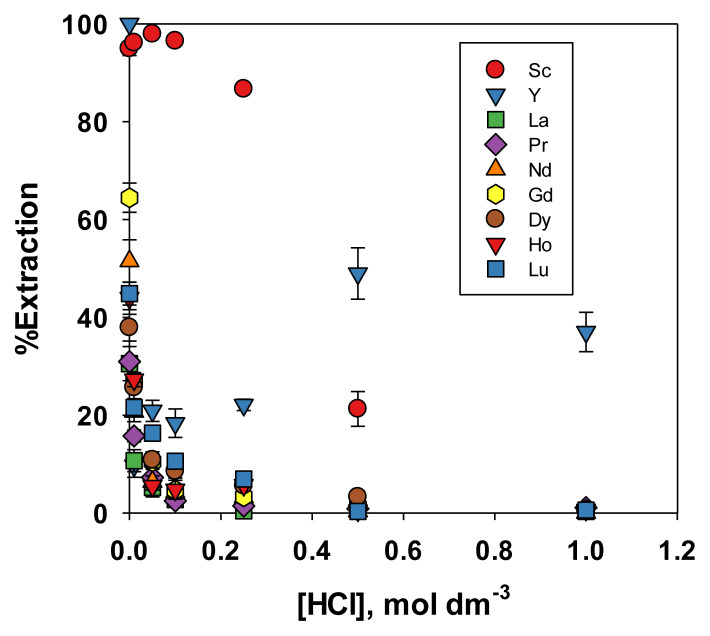
Effect of HCl concentration on the extraction efficiency of REEs.

**Figure 4:**
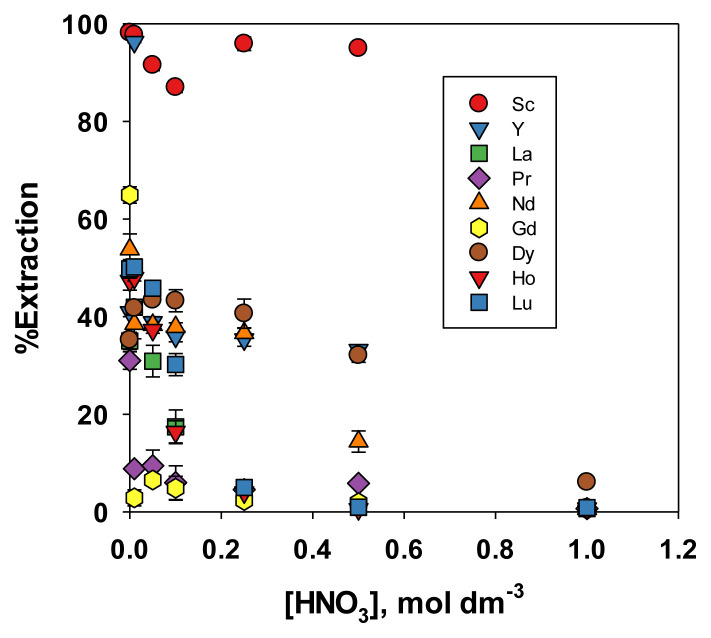
Effect of HNO_3_ concentration on the extraction efficiency of REEs.

The results in Figure 2 showed that Sc was selectively extracted from a solution also containing Nd, and Dy dissolved in 0.1 mol dm^−3^ HCl. Therefore, the selectivity of Sc extraction compared with other REEs at various HCl concentrations must be verified. In the preliminary study [18], HCl and HNO_3_ were used as the media for the extraction of Nd, Dy, and Ni. In this expanded study, these two media were used to extract Nd and Dy as well as other REEs including Sc, yttrium (Y), lanthanum (La), praseodymium (Pr), Nd, gadolinium (Gd), Dy, holmium (Ho), and lutetium (Lu). HCl and HNO_3_ were generally used in leaching REEs from secondary products such as phosphogypsum [19–21] and waste permanent magnets [22,23]. Therefore, these two kinds of acid at various concentrations were used as the extraction medium, and the results of the experiment were shown in Figures 3 and 4. Figure 3 showed that increasing the HCl concentration reduced the extraction percentage of all tested REEs. As explained in the previous paper [18], this occurred due to the conversion of the PIL (R’COO^−^ + NH(R)_3_) into quaternary ammonium chloride and decanoic acid upon contact with HCl. Figure 3 also showed that HCl at a concentration between 0.01 to 0.25 mol dm^−3^ was appropriate for extracting Sc with a PIL composed of decanoic acid and TOA. Within this concentration range, the extraction efficiency of Sc ranged from 86% to 96%, while that of other REEs remained below 27%, except for Y, which reached 44% at an HCl concentration of 0.01 M. In addition, the relatively high extraction efficiency of Y is understandable, as its atomic size was larger than that of Sc but smaller than those of other REEs. This showed that these conditions were suitable for selective extraction of Sc. When HNO_3_ was used as the extraction medium, the trend was similar to that observed with HCl, as shown in Figure 4. Sc showed the highest extraction efficiency, followed by Y. However, the extraction efficiency of the other REEs was higher (±85% for Y and <50% for others) with the HNO_3_ extraction medium than with the HCl extraction medium, nitrate ions were more easily coextracted with the metal ions due to the high chaotropic activity of nitrate compared to chloride [18]. Consequently, selective extraction of Sc was effectively achieved using HCl at lower concentrations (0.01 to 0.25 mol dm^−3^) as the extraction medium, compared with HNO_3_.

#### 3.2.3 Effect of salt concentration on REE extraction

**Figure 5:**
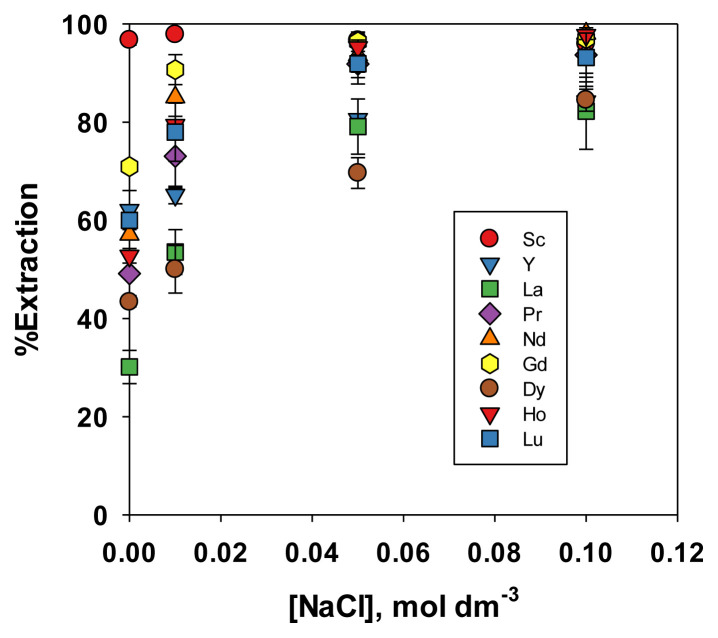
Effect of NaCl concentration on the extraction efficiency of REEs.

**Figure 6:**
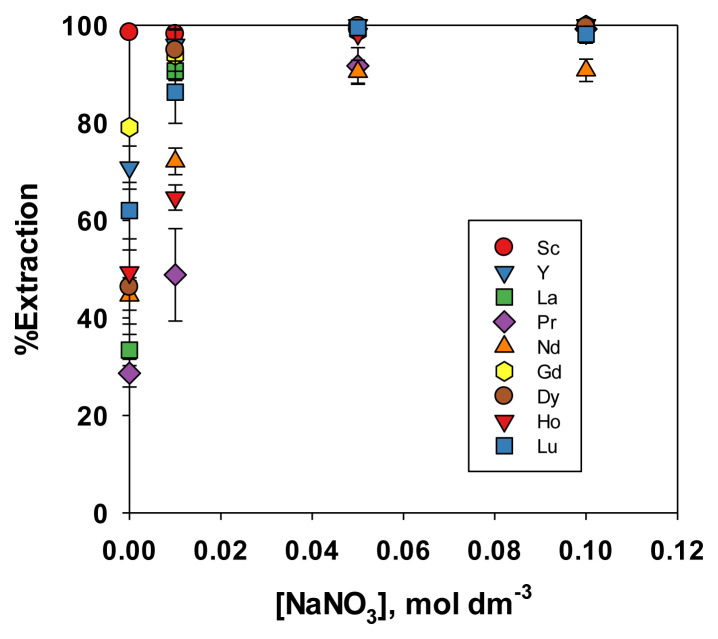
Effect of NaNO_3_ concentration on the extraction efficiency of REEs.

A previous paper [18] also observed that NaCl and NaNO_3_ were preferred as media for selectively extracting REEs from Ni. This study investigated the use of these media in the extraction of a wider range of REEs, with the results presented in Figures 5 and 6. Similar to the experiment with acid media, Sc exhibited the highest extraction efficiency, specifically at a lower salt concentration. However, the extraction efficiencies of other REEs were very close to that of Sc. Although all nine REEs were evaluated, at high salt concentrations (0.1 mol dm^−3^), their extraction percentage converged to similar values. Due to significant marker overlap on the plot, only five and three points being distinguishable in Figure 5 and 6 repectively. In NaCl or NaNO_3_ media, REEs formed stable complexes with Cl^−^ or NO_3_^−^, which were either more soluble in the organic phase or more reactive with the extractant. As the concentration of NaCl or NaNO_3_ increased, all REEs were more likely to form such complexes, thereby reducing the difference in extraction efficiency among them. The extractant tended to coextract all REEs in totality, and the selectivity gradually became lower [32]. Therefore, this extraction condition was only suitable for the collective extraction of REEs, specifically at a high concentration of NaCl or NaNO_3_, and was not useful for selective extraction. This was because the rare earth metals were extracted by the salting-out effect, which was not highly selective.

#### 3.2.4 Effect of volume ratio

**Figure 7:**
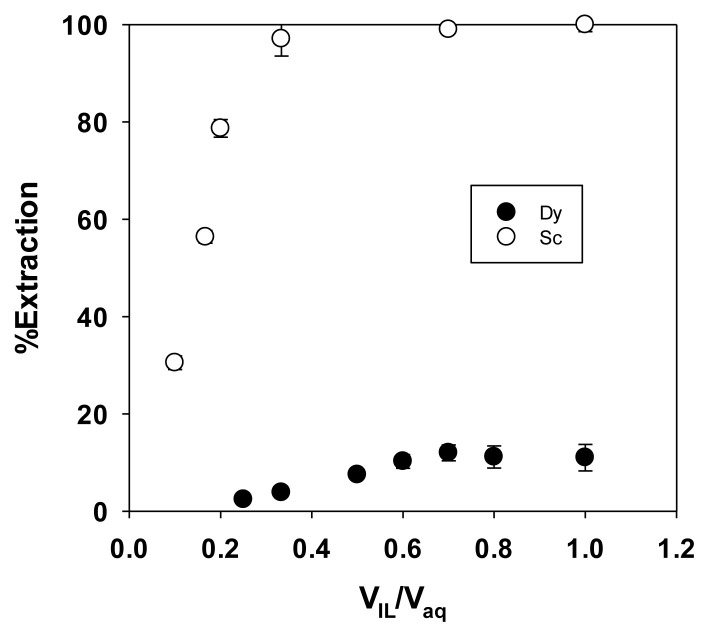
Effect of organic-to-aqueous volume ratio on the extraction efficiency of Sc.

The organic-to-aqueous volume ratio was an important factor for extraction conditions [33]. Using too much organic phase was not economical while using too little could prevent maximum extraction. The effect of the volume ratio on the extraction efficiency of Sc and Dy using 0.1 mol dm^−3^ HCl as the extraction medium was shown in Figure 7, where the HCl medium selectively extracts Sc over Dy. Almost all Sc in the aqueous phase was extracted into the organic phase at an organic-to-aqueous volume ratio of 0.7. Under these conditions, approximately 17% of Dy was extracted, confirming that this PIL was a selective extractant for Sc.

To evaluate the performance of the synthesized PIL made from decanoic acid and TOA, its selectivity and extraction efficiency for Sc were compared with several reported extractant systems in the literature. These included synergistic extractants such as HTTA-TOPO [34], D2EHAG/D2EHAF [35], and Cyanex272/Cyanex923 [36] and ILs such as [N333MeOAc][Tf_2_N] [37]. Table 2 presented the comparison of Sc extraction efficiencies and selectivities measured in this study with those from previous work. The cited studies used acidic sulfate or nitrate medium and obtained efficiencies of Sc extraction greater than 80% but with different coextraction modes that depended on extractant chemistry and diluent used. Along with its high efficiency, the PIL system also benefited from excellent Sc selectivity. Unlike synergistic systems that incorporated multiple extractants, the PIL was a single-component system under moderately acidic conditions (0.1–0.5 mol dm^−3^ HCl). Its easy synthesis, no flammable organic diluents, and convenience of operation placed it as an appealing candidate for scalable and green rare earth separations.

## 4. Conclusion

In conclusion, this study unveiled the first-ever use of a hydrophobic PIL composed of decanoic acid and TOA to selectively extract Sc from other REEs using acid or salt as the extraction media, which produced different extraction effects. The concentration of salt directly corresponded to the extraction efficiency of REEs, while the concentration of acid had the opposite effect. Salt media provided a high extraction percentage for all REEs tested, making it suitable for the collective extraction of REEs, particularly at high salt concentrations. HCl media exhibited higher selectivity for Sc extraction, specifically at low HCl concentrations. This study observed that maximum Sc extraction was achieved at an organic-to-aqueous phase volume ratio of 0.7. Under these conditions, Sc was almost completely extracted while Dy extraction remained below 17%.

## Figures and Tables

**Scheme:**
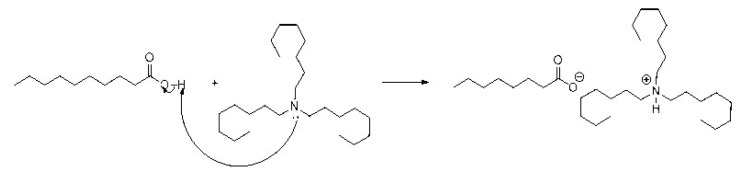
Formation mechanism of PIL from decanoic acid and tri-octylamine.

**Table 1:** Viscosities (mPa•s) of synthesized PILs at 30 °C.

		Dodecanoic,	Decanoic,	Octanoic,	Heptanoic,
		solid	solid	6.27 ± 0.19	5.65 ± 0.11
TOA	7.67 ± 0.19	20.90 ± 0.06	17.52 ± 0.06	15.68 ± 0.15	10.14 ± 0.46
THA	4.18 ± 0.19	15.10 ± 0.19	13.39 ± 0.11	11.54 ± 0.11	10.14 ± 0.11

**Table 2:** Comparison of Sc extraction efficiencies and selectivities in this study and previous reports.

Extractants	Extractant diluent	Aqueous Media	%E		Ref.

			Sc	Other Cations	
Sinergistic: HTTA-TOPO	n-dodecane	H_2_SO_4_ & (NH_4_)_2_SO_4_, pH ≥3	100	Fe (100), Co≈Mn (< 80), Zn (< 60), Ni (< 40), Al(≈0)	[34]
D_2_EHAG & D_2_EHAF	n-dodecane	Sulfate (pH 2)	100	Fe(100), Ni (<30), Co, Mn, Al, Cr, Ca, Mg (<10)	[35]
Sinergistic: Cyanex272 and Cyanex923	n-heptane	H_2_SO_4_ (0.10–1.05 mol/L)	< 90	-	[36]
[N_333_MeOAc][Tf_2_N]	chloroform	Nitrate pH 5	83.4	Other REEs (<30)	[37]
[N_444_ MeOAc][Tf_2_N]			95.9		
Sinergistic: D2EHPA/TBP	Sulfonated kerosene	Sulfate	>99	Al, Fe, Ti, La, Ce, Ca (<5)	[23]
PIL	No dilution	HCl (0.1–0.25 M)	>99	Y, La, Pr, Nd, Gd, Dy, Ho, Lu (<20)	This work
